# Phenolic Extract from Extra Virgin Olive Oil Induces Different Anti-Proliferative Pathways in Human Bladder Cancer Cell Lines

**DOI:** 10.3390/nu15010182

**Published:** 2022-12-30

**Authors:** Carmela Spagnuolo, Stefania Moccia, Idolo Tedesco, Giuseppina Crescente, Maria Grazia Volpe, Maria Russo, Gian Luigi Russo

**Affiliations:** Institute of Food Sciences, National Research Council, 83100 Avellino, Italy

**Keywords:** extra virgin olive oil phenols, bladder cancer, apoptosis, autophagy, antioxidant effect

## Abstract

Regular consumption of olive oil is associated with protection against chronic-degenerative diseases, such as cancer. Epidemiological evidence indicates an inverse association between olive oil intake and bladder cancer risk. Bladder cancer is among the most common forms of cancer; in particular, the transitional cell carcinoma histotype shows aggressive behavior. We investigated the anti-proliferative effects of a phenolic extract prepared from an extra virgin olive oil (EVOOE) on two human bladder cancer cell lines, namely RT112 and J82, representing the progression from low-grade to high-grade tumors, respectively. In RT112, the EVOOE reduced cell viability (IC50 = 240 μg/mL at 24 h), triggering a non-protective form of autophagy, evidenced by the autophagosome formation and the increase in LC-3 lipidation. In J82, EVOOE induced a strong decrease in cell viability after 24 h of treatment (IC50 = 65.8 μg/mL) through rapid and massive apoptosis, assessed by Annexin V positivity and caspase-3 and -9 activation. Moreover, in both bladder cancer cell lines, EVOOE reduced intracellular reactive oxygen species, but this antioxidant effect was not correlated with its anti-proliferative outcomes. Data obtained suggest that the mixture of phenolic compounds in extra virgin olive oil activates different anti-proliferative pathways.

## 1. Introduction

Bladder cancer is the 10th most common form of cancer worldwide and is primarily a disease of the elderly. The incidence and mortality rates in men are 9.5 and 3.3 per 100,000, about 4 times less in women [1]. Incidence rates are highest in Southern Europe (Greece; Spain; Italy), Western Europe (Belgium and The Netherlands), and Northern America. The main risk factor for bladder cancer is considered occupational exposure to chemicals, water contaminants, and cigarette smoking [2,3]. Urothelial cell carcinoma (UCC) is the most common form of bladder cancer, accounting for more than 90% of diagnosed cases [4]. UCC begins as superficial bladder carcinomas and progresses heterogeneously with a variable course. As reported by European guidelines, about 75% of patients with bladder cancer have a disease confined to the mucosa (non-invasive papillary carcinoma Ta stage, carcinoma in situ Tis) or submucosal (T1 stage) [5]. The pathological stage is an important prognostic factor that is critical for patient management. Histological grading of urothelial non-muscle-invasive bladder urothelial carcinomas is classified according to the WHO 1973 (as grade 1–3) and/or the WHO 2004/2016 (papillary urothelial neoplasm of low malignant potential, low grade/high grade) systems [5]. The low-grade/G1 tumors show a low progression rate and generally only require endoscopic treatment and surveillance. The high-grade/G3 tumors have significant malignant potential with significant progression and high cancer mortality rates [6].

The diet may play a considerable role in bladder carcinogenesis, considering that many food metabolites are excreted through the urinary tract. Epidemiological evidence showed that a healthy diet (defined by commonly used dietary scores) might be protective against the risk of invasive UCC, especially in current and former smokers [7]. In particular, some epidemiological studies reported that high adherence to the Mediterranean diet showed significantly decreased bladder cancer risk [8,9]. Similarly, in a pooled analysis of 13 cohort studies, the Mediterranean diet was associated with a reduced risk of developing bladder cancer [10]. The importance of a healthy diet has been further confirmed by studies that highlighted the positive correlation between red or processed meat intake and bladder cancer risk, confirming the hypothesis that, as opposed to a Mediterranean diet, Western dietary patterns may contribute to the etiology and prognosis of bladder cancer [11,12].

It has also been reported a potential role of specific foods against bladder cancer risk [3]. Among these, according to a Belgian case-control interventional study, regular consumption of olive oil showed a chemopreventive effect on bladder cancer risk, compared to an increased risk associated with a high intake of cheese [13].

Extra Virgin Olive Oil (EVOO) is generally considered a key contributor to the healthy aspects attributed to the Mediterranean diet [14,15,16]. It is obtained by the cold-pressing method of the olive fruit and is composed of a glycerol fraction (95–99%) and a non-glycerol or unsaponifiable fraction (0.4–5%) that contains the phenolic fraction [17]. More than 30 phenolic compounds have been identified in EVOO. Their varieties, chemical structures, and specific concentrations differ depending on several factors, including cultivars, growing region, agricultural techniques, maturity of the olive fruit at harvest, and processing [18,19]. The main classes of EVOO polyphenols are secoiridoids, phenylethanoids, flavonoids, phenolic acids, hydroxy-isocromans, and lignans [14,18]. These phenolic components strongly contribute to the health effects attributed to EVOO, possessing, among others, anti-inflammatory, antioxidant, and anti-microbial properties [20].

Chemopreventive properties of EVOO and its phenolic compounds have emerged from epidemiological studies [13,21]. In vitro and in vivo data describe the EVOO polyphenols’ capacity to suppress, arrest, or reverse carcinogenesis by acting as cancer-blocking and/or cancer-suppressing agents [22,23]. In general, the former prevents the harmful action of carcinogens by several mechanisms, including enhancing carcinogen detoxification, modifying carcinogen uptake and metabolism, scavenging ROS (reactive oxygen species) and other oxidative species, and enhancing DNA repair; the latter inhibits cancer promotion and progression after the formation of pre-neoplastic cells [24,25]. In this context, the potent antioxidant properties of EVOO phenolics may provide considerable protection against cancer, especially in the early stages [26,27]. Moreover, beyond this aspect, phenolic compounds can also modulate molecular pathways regulating different stages leading to oncogenesis [28,29,30]. According to the EPIC study, an inverse association between the dietary intakes of flavonoids (mainly flavonols) and lignans versus the risk of bladder cancer, particularly the aggressive UCC form [31]. Considering that most of the flavonoid and lignan metabolites are excreted through urine, exposing the surfaces of the bladder to them, their potential chemopreventive action against bladder cancer is plausible.

A few in vitro studies support the mentioned epidemiological evidence, highlighting the chemopreventive activity of polyphenols extracted from EVOO on bladder cancer cells. In particular, the extracts examined were able to suppress migration and invasion, modulate the matrix metalloproteinase-2 [32], block cell cycle progression, and modulate chemotherapeutic toxicity [33]. Other in vitro and in vivo studies evidenced the potential activity of pure flavonoids present in EVOO against bladder cancer. As an example, apigenin is capable of reducing cell proliferation, blocking cell cycle progression, and inducing apoptosis [34]. In addition, apigenin exerts anti-invasion effects acting on (ERK1/2, JNK)/AP-1 and (ERK1/2, JNK)/NF-κB signaling pathways and, consequently, inhibiting the urokinase-type plasminogen activator receptor (uPAR) expression in human T24 cells [35]. Another example is luteolin, which suppresses bladder cancer growth (in vitro and in vivo) by upregulating p21 and inhibiting mTOR signaling [36].

The present work aims to study the chemopreventive effects of an Extra Virgin Olive Oil Phenolic Extract (EVOOE) in bladder cancer cell lines characterized by different tumor progression stages and verify if a functional link may exist between the EVOOE antioxidant properties and its capacity to modulate cell growth. To these purposes, two cell lines representative of epithelial- and mesenchymal-like bladder carcinoma cells were used: RT112 as a model of moderately differentiated (grade 2) transitional cell carcinoma, and J82 that represents a more aggressive and poorly differentiated (grade 3) transitional cell carcinoma [37,38].

## 2. Materials and Methods

### 2.1. Reagents

RT112 and J82 cell lines from ATCC were available at the Institute of Food Science, Avellino, Italy; Roswell Park Medium Institute (RPMI) and Minimum Essential Medium Eagle (EMEM) medium, fetal bovine serum (FBS), L-glutamine 200 mM, penicillin 5000 IU/mL/streptomycin 5000 μg/mL, and PBS (phosphate buffer saline) were purchased from Lonza (Euroclone SPA, Pero, Italy); Crystal Violet, trypan blue solution (0.4% *v*/*v*), RNase A, propidium iodide, quercetin, rutin, myricetin, gallic acid, kaempferol and dimethylsulfoxide (DMSO) were from Merck Life Science (Milano, Italy). Glutathione (GSH); phtaldialdehyde, 2′,7′-dichlorofluorescein diacetate (DCFH-DA), *N*-acetyl-L-cysteine (NAC), L-Buthionine-sulfoximine (BSO) were from Carlo Erba (Milan, Italy). Reagents for Folin–Ciocalteau were purchased from Merck Life Science (Milan, Italy). Methanol, water (HPLC-grade), and trifluoroacetic acid were purchased from Merck-Schuchardt (Hohenbrunn, Germany). Hydroxytyrosol, tyrosol, oleocanthal, oleuropein aglycone, and ligstroside aglycone analytical standards were purchased from Merck-Schuchardt (Hohenbrunn, Germany).

### 2.2. Phenolic Compound Extraction from Extra Virgin Olive Oil

The EVOO, an Italian blend (Sud Italia 637), was generously provided by Basso Fedele & Figli s.r.l (San Michele di Serino, Avellino, Italy). Phenolic compounds from EVOO were isolated following the analytical procedure [26]. Briefly, 10 g of oil was homogenized for 3 min in a solution of 80% methanol (10 mL) and centrifuged at 4000× *g* for 15 min. The procedure was repeated three times. A de-fatting procedure using *n*-hexane was performed to eliminate the lipid fraction. Aliquots (1 mL) of the raw hydrophilic extract were lyophilized and stored at −80 °C until use. The extract (EVOOE) was re-suspended in DMSO at a stock concentration of 40 mg/mL immediately before the experiments.

The total phenol content of EVOOE was determined by Folin–Ciocalteu’s method, as reported in [39], and data were expressed as mg of gallic acid equivalent (GAE)/100 g of oil. Briefly, a solution of Folin reagent (1/20), 2% Na_2_CO_3_, the sample (1/100) and water was prepared and incubated at 37 °C for 2 h. Subsequently, absorbance was measured at 760 nm (Synergy HT microplate reader, BioTek, Milan, Italy).

### 2.3. HPLC-UV-DAD Analysis

The analysis was performed on a 1260 Infinity II LC System (Agilent, Santa Clara, CA, USA) equipped with an Agilent G7111A quaternary pump and a WR G7115A diode array detector. The separation was done with Poroshell 120 EC-C18 (150 × 4.6 mm i.d., 4.0 μm particle size, Agilent, Santa Clara, CA, USA) column at 30 °C, using water (mobile phase A) and acetonitrile (mobile phase B), both with 0.02% trifluoroacetic acid. The elution condition involved a linear gradient as follows: 0–2.5 min, 5→20% B; 2.5–5 min, 20→30% B; 5–12 min, 30→45% B; 12–17 min, 45→60% B; 17–21 min, 60→80% B; held at 80% B for other 6 min. Phase B reached 95% and held at 95% for 3 min; then returned to the starting conditions and re-equilibrated for 2 min. The total analysis time was 32 min, the flow rate was 0.5 mL/min, and the injection volume was 20 μL. UV detection was set at four different wavelengths (220, 280, 320, and 360 nm). Identification was carried out by comparing the retention times and spectral data with those of standards.

### 2.4. Cell Culture and Viability Assay

RT112, human bladder carcinoma epithelial cells [40], and J82, human urinary bladder transitional mesenchymal carcinoma cells [41], were cultured in RPMI and MEM, respectively, supplemented with 10% FBS, 1% L-glutamine, 1% penicillin/streptomycin, at 37 °C in a humidified atmosphere containing 5% CO_2_. Cell viability was assayed by Crystal Violet staining. Briefly, 8 × 10^4^/mL cells were seeded in 48 well plates and stimulated with different concentrations of the extract at the indicated times. Then cells were fixed with 10% formalin for 10 min, washed, and subsequently Crystal Violet (0.1% *w*/*v*) was added for 30 min. Finally, cells were lysed with 10% acetic acid, and the absorbance was spectrophotometrically measured at 590 nm.

### 2.5. Autophagy Detection

Autophagy was assessed by measuring autophagic vacuoles and evaluating the expression of the lipidated isoform of LC3-II protein.

#### 2.5.1. Measurement of Autophagic Vacuoles

The Cyto-ID Autophagy Detection Kit (ENZO Life Science, Milan, Italy) was used to monitor autophagy following the manufacturer’s protocol. Briefly, RT112 cells were stimulated for 24 h with EVOOE, washed, and incubated with the autophagy detection marker (Cyto-ID). Subsequently, cells were washed with assay buffer and photographed using a fluorescence microscope (Zeiss Axiovert 200, Carl Zeiss, Milan, Italy). Autophagosomes were detected by flow cytometry (FACS-Calibur; Becton Dickinson, Mountain View, CA, USA) equipped with an argon laser (488 nm) and filtered at 530 nm, and analyzed using CellQuest software (Becton Dickinson, Mountain View, CA, USA).

#### 2.5.2. Immunoblottings

RT112 cells were incubated with EVOOE as indicated and, at the end of stimulation, were lysed using a lysis buffer containing protease and phosphatase inhibitors, as reported [42]. Following protein concentration determination [43], 30 μg of protein lysates were loaded on a 4–12% precast gel (Novex Bis-Tris precast gel 4–12%; Life Technologies), and the MES (2-(*N*-morpholino) ethanesulfonic acid) buffer was used. The primary antibodies used were: anti-LC3 (Cell Signalling Technology, Milan, Italy; cat #12741) and anti-α-tubulin (Merck Life Science, Milan, Italy; cat #T9026). PVDF membranes were incubated with horseradish peroxidase-linked secondary antibody raised against mouse and immunoblots developed using the ECL Plus Western blotting detection system kit (GE Healthcare, Milan, Italy). The measurement of the optical density was performed on a Gel Doc 2000 Apparatus (Bio-Rad Laboratories, Milan, Italy), and Multianalyst software (Bio-Rad Laboratories, Milan, Italy) was used to quantify band intensities.

### 2.6. Apoptotic Assays

To verify the induction of apoptosis, three different assays were used: detection of apoptotic bodies, Annexin V exposure, and caspase-9 and -3 enzymatic activities.

#### 2.6.1. Hoechst Staining

J82 cells, 0.15 × 10^6^/mL in 6-well plates, were incubated with EVOOE for 15 h. After incubation, cells were washed and added with the nuclear dye Hoechst 33,342 (1 μg/mL). Apoptotic nuclei were visualized using fluorescent microscopy and photographed in a DAPI filter.

#### 2.6.2. Annexin V Detection

Phosphatidylserine (PS) externalization was assessed using the fluorescein-isothiocyanate-labeled (FITC) Annexin V, which binds PS, as indicated in the manufacturer’s protocol (Miltenyi Biotec, Bologna, Italy). Briefly, treated J82 cells (0.15 × 10^6^/mL) were collected and suspended in 100 μL of binding buffer with Annexin V FITC (10 μL) and incubated in the dark at room temperature; after centrifugation, cells were suspended in 500 μL of binding buffer and 25 μg/mL of propidium iodide before flow cytometry acquisition. A total of 10,000 events were collected, and low fluorescence debris and necrotic cells were gated out before the analysis was performed using CellQuest software (Becton Dickinson, Mountain View, CA, USA).

#### 2.6.3. Caspases Assay

For caspase-9 and -3 enzymatic activities, J82 cells (0.15 × 10^6^/mL) were incubated for 6 h, as described in the figure legends. After stimulation, the cells were washed in PBS and suspended in lysis buffer (10 mM HEPES, pH 7.4; 2 mM ethylenediaminetetraacetic acid; 0.1% 3-((3-Cocamidopropyl) dimethylamino)-1-propane sulfonate; 5 mM dithiothreitol; 1 mM phenylmethylsulfonylfluoride; 10 μg/mL pepstatin-A; 10 μg/mL aprotinin; 20 μg/mL leupeptin). Cell extracts with reaction buffer and the respective conjugated amino-4-trifluoromethyl coumarin (AFC) substrates, benzyloxycarbonyl-Asp(OMe)-Glu(OMe)-Val-Asp(OMe)-AFC(ZDEVD-AFC) for caspase-3 and LEHD-AFC for caspase-9 (carbobenzoxy-Asp-Glu-Val-Asp and Leu-Glu-Hys-Asp-AFC) were incubated at 37 °C for 30 min. The free fluorochrome AFC was detected with excitation and emission setting of 395 ± 20 and 530 ± 20 nm, respectively (Synergy HT microplate reader, BioTek, Milan, Italy). An AFC standard curve was determined to quantify the enzymatic activities. Caspase-specific activities were calculated as nmol of AFC produced per minute per mg of proteins at 37 °C at saturating substrate concentrations (50 μM). Fold increase in caspase-3 and -9 activities was determined in comparison with the level of control cells.

### 2.7. Intracellular ROS Measurement

RT112 and J82 cells, 0.01 × 10^6^/mL in 96-well plates, were treated with EVOOE as reported and subsequently incubated for 30 min with 10 μM of 2′-7′-dichlorofluorescein diacetate (DCFH-DA). The diacetate group of DCFH-DA is hydrolyzed by cellular esterase, and DCFH is oxidized to a fluorescent molecule 2′-7′-dichlorofluorescein (DCF) by intracellular peroxides. After stimulation, the cells were washed twice with PBS and then fluorescence was assessed by a spectrofluorometer with an excitation and emission setting of 485 ± 20 nm and 530 ± 20 nm, respectively.

### 2.8. Glutathione Determination

After treatment with EVOOE for 3 h, RT112 and J82 cells (0.15 × 10^6^/mL) were collected, pellets were washed with PBS, and subsequently, proteins were precipitated with trichloroacetic acid (5% *v*/*v* final concentration in 0.1 M HCl and 10 mM EDTA). The fluorescence of the supernatant was measured, using phthaldialdehyde as substrate, at an excitation and emission setting of 340 ± 20 nm and 460 ± 20 nm, respectively. The concentration of GSH was extrapolated from a standard curve calculated using pure GSH and expressed as a percent of untreated cells.

### 2.9. Statistical Analysis

Data are expressed as the mean ± standard deviation (s.d.) or, to consider the sample size, mean ± standard error (s.e.) and analyzed by Student’s t-test to evaluatef the significance of the single treatment vs. control.

## 3. Results

### 3.1. Identification of the Compounds in EVOOE by HPLC-UV-DAD

In the present study, we investigated, through a targeted approach, the presence of the most abundant compounds which are reported to exist in EVOO, according to already published studies.

**Figure 1:**
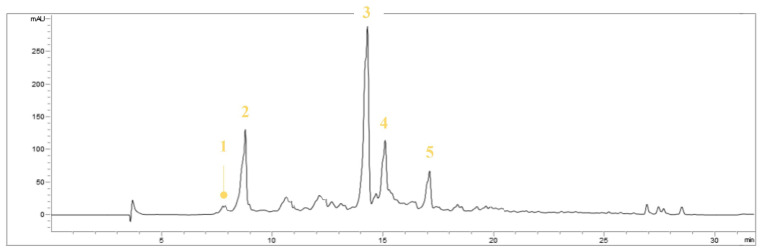
HPLC-UV recorded chromatogram at 280 nm of EVOOE. Compounds were numbered, based on their increasing retention time, as follow: hydroxytyrosol (compound 1), tyrosol (compound 2), oleocanthal (compound 3), oleuropein aglycone (compound 4), and ligstroside aglycone (compound 5).

In detail, the chromatographic separation of the olive oil polyphenolic extract by HPLC-UV-DAD revealed the presence of tyrosol (Tyr), oleocanthal (OC), oleuropein aglycone, and ligstroside aglycone as major components, together with other minors (Figure 1). Among these, benzoic acid and apigenin have been detected. Identification was based on retention time, UV-visible, and pure analytical standards.

**Figure 2:**
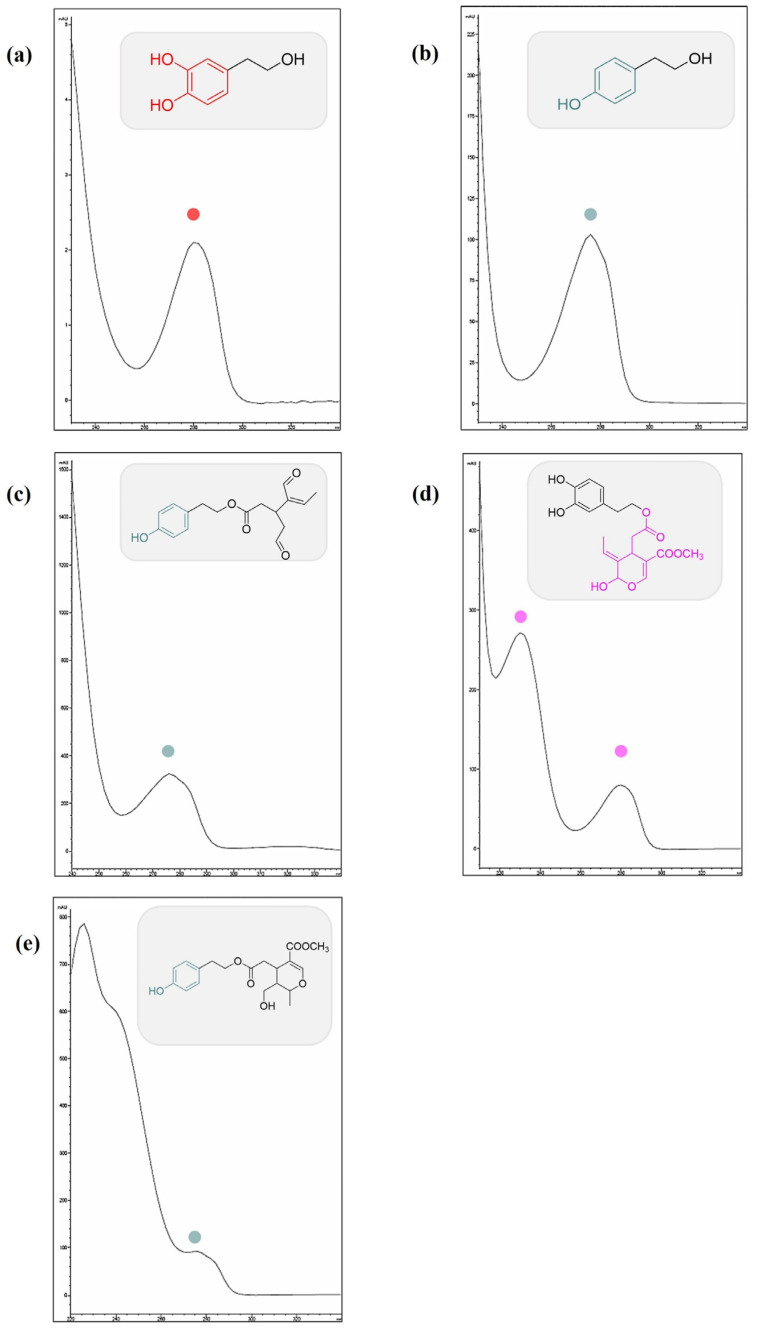
UV-DAD spectra of compounds 1 hydroxytyrosol (**a**), 2 tyrosol (**b**), 3 oleocanthal (**c**), 4 oleuropein aglycone (**d**), and 5 ligstroside aglycone (**e**) identified in EVOOE. In the grey panels, the structures of each compound are reported.

Compounds (**1**) and (**2**) were identified as hydroxytyrosol and tyrosol, respectively. The discrimination of these compounds was possible not only based on their retention time but also the different chromophoric moieties. Hydroxytyrosol possesses catechol as a chromophoric moiety, and the UV spectrum showed a λ_max_ at 280 nm; instead, the UV spectrum of tyrosol with phenol as a chromophoric moiety showed a λ_max_ at 275 nm (Figure 2a,b). Oleocanthal (**3**), a tyrosol derivative, has the same UV-visible spectrum as its precursor and has the same chromophoric moiety (phenol); in detail, the UV spectrum showed a λ_max_ at 275 nm, with a shoulder at 280 nm (Figure 2c). Oleocanthal does not occur in the plant *Olea europaea* L. (leaves and fruits) but is formed during EVOO manufacturing by the conversion of oleuropein and ligstroside [44]. It is the molecule responsible for the “burning in the throat” or the spicy sensation we feel when we ingest EVO oil [45].

The UV spectrum of compound (**4**), identified as oleuropein aglycone, showed two absorption peaks at 230 and 280 nm, both in the UV region: the absorption at 230 nm is due to the unsaturated ester group, while that one at 282 nm of the dihydroxy phenyl group (Figure 2d) [46,47]. Compound (**5**) was tentatively identified as ligstroside aglycone. It is among the most abundant phenols present in extra-virgin olive oil and derives from tyrosol, and elenolic acid [48]; shares with tyrosol the occurrence of phenol as a chromophore moiety with a λ_max_ at 275 nm. In addition, a maximum UV absorption at 235 nm was also detectable (Figure 2e).

In virgin olive oil, secoiridoid aglycons constitute an important class of phenolic compounds and are genetically related to oleuropein and ligstroside [49].

### 3.2. Extra Virgin Olive Oil Phenolic Extract Stimulation Reduces Cell Viability in RT112 and J82 Bladder Cell Lines

The total phenolic content of the EVOOE used in the present work was 19.8 mg GAE/100 g, as measured by Folin–Ciocalteu’s reagent. This value was in line with the one reported in several publications [50,51,52] and near the range reported by the Phenol-Explorer database, i.e., 55.14 ± 23.5 mg/100 g [53].

**Figure 3:**
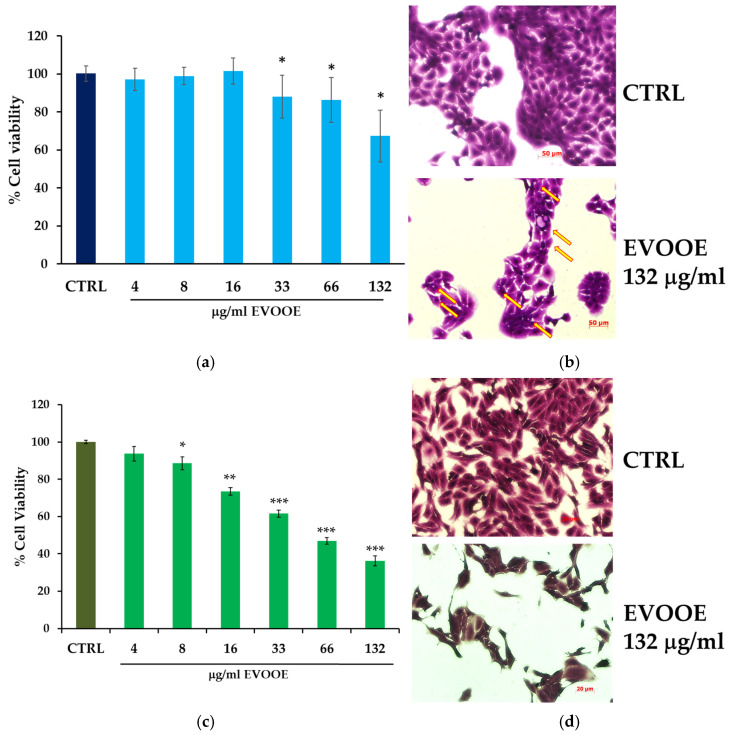
EVOOE stimulation effects on cell viability in RT112 and J82 cells. RT112 (**a**) and J82 (**c**) cells were stimulated with the indicated concentration of EVOOE (*w*/*v*) for 24 h. Cell viability was evaluated by Crystal Violet assay. Bar graphs represent the mean of three experiments (±s.e.). Symbols indicate significance: *p* < 0.05 (*), *p* < 0.0005 (**), and *p* < 0.0001 (***) with respect to CTRL (DMSO-treated cells). Panels (**b**,**d**) report representative images of RT112 and J82 cells, respectively, untreated (left) and treated (right) with the indicated concentrations of EVOOE and stained with Crystal Violet (optical microscope Axiovert 200 M Zeiss; 400× or 200×, bright field). The arrows in the lower panel in (**b**) indicate the presence of intracellular vacuoles.

To study the effects of EVOOE on different phases of bladder cancer progression, two cell lines were employed, RT112 and J82, representing, respectively, low- and high-grade tumors. In order to assess the anti-proliferative effect of the EVOOE, cells were treated for 24 h within a range of concentrations corresponding to 4–132 μg/mL (*w*/*v*) of EVOOE (Figure 3a,c). The treatment slightly reduced the amount of viable RT112 cells (Figure 3a,b), with the higher concentration, 132 μg/mL, that induced about a 30% decrease. On the contrary, J82 cells showed a rapid and strong response to EVOOE (Figure 2c,d), with a 40% decrease in cell viability at the concentration of 33 μg/mL. The calculated IC_50_ values were 240 μg/mL and 65.8 μg/mL for RT112 and J82 cells, respectively.

It is known that phenolic compounds may generate hydrogen peroxide through their interaction with culture media components, causing potential confounding effects on cell growth [54]. To exclude this artifactual phenomenon, we incubated EVOOE with MEM and RPMI medium at the same time and concentrations used for the cell line experiments, verifying by the FOX assay method [55] that the extract did not generate a significant amount of hydrogen peroxide that could interfere with cell growth (Table S1).

### 3.3. Extra Virgin Olive Oil Phenolic Extract Induces Autophagy in RT112 Cell Line

**Figure 4:**
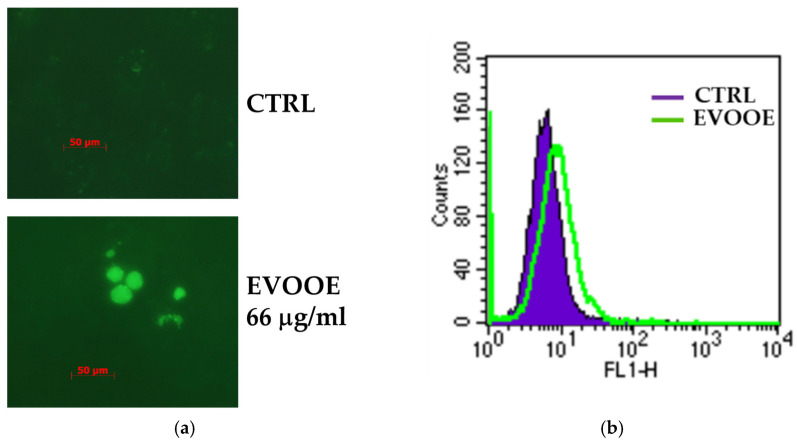

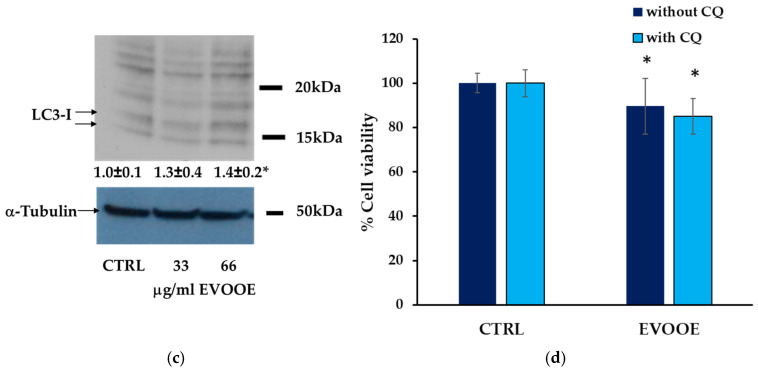
EVOOE induces autophagy in RT112 cells. (**a**) Representative images of cells untreated (top) and treated with EVOOE 66 μg/mL for 24 h (bottom) following staining with CytoID. Autophagic vacuoles were visualized using fluorescent microscopy and photographed in a FITC filter with 200× magnification. (**b**) Representative histogram of CytoID flow cytometry acquisition of cells untreated (blue) and treated with EVOOE 66 μg/mL for 24 h (green line). (**c**) Immunoblotting analysis of LC3-I/LC3-II expression in RT112 cells treated for 24 h with EVOOE. Blots are representative of one out of three separate experiments performed. Densitometric analysis (numbers between panels) is expressed as the ratio between LC3-II, and α-tubulin band intensities mean ± s.d. with symbols that indicate significance: *p* < 0.05 (*) compared to CTRL. (**d**) RT112 cells were treated for 24 h with the indicated concentration of 66 μg/mL EVOOE in a single treatment or following a pre-incubation for 1 h with the autophagic inhibitor chloroquine (CQ; 20 μM). Cell viability was evaluated by the Crystal Violet assay. Bar graphs represent the mean of three experiments (±s.d.). Symbols indicate significance: *p* < 0.05 (*) compared to CTRL.

After 48 and 72 h of treatment, the reduction in viability induced by EVOOE in RT112 cells was of the same magnitude as that at 24 (Figure S1). In the attempt to understand the mechanism(s) responsible for the EVOOE anti-proliferative effects, we observed that the reduced cell proliferation in the RT112 cell line was neither associated with cell cycle arrest nor cell death. The presence of intracellular vacuoles in EVOOE-treated cells, which emerged by microscopy observation (as indicated by the arrows in Figure 3b), suggested the possible activation of an autophagic process. Multiple assays were carried out to detect autophagy to verify this hypothesis [56]. To help us to visualize and quantify the autophagosomes, RT112 cells stimulated with EVOOE were stained with Cyto-ID Green autophagy dye. Figure 4a,b shows fluorescent autophagic vacuoles, which increase by about 30% compared to untreated cells, indicating the activation of autophagy obtained by stimulating cells with EVOOE 66 μg/mL for 24 h (clearly visible by fluorescence microscopy, Figure 4a), quantified by flow cytometry (Figure 4b). To further confirm the autophagy activation induced by EVOOE, we assessed the modulation of LC3-II, the lipidated isoform of LC3 protein, a molecular marker essential in autophagosome membrane formation [56]. The immunoblots and the corresponding densitometric analysis, reported in Figure 4c (numbers between panels), show a significantly increased expression of the LC3-II band after 24 h of incubation with EVOOE.

Furthermore, we studied which form of autophagy was induced by EVOOE in RT112 cells. Excluding cytotoxic and cytostatic autophagy, characterized respectively by cell death and cell cycle arrest (both processes were undetectable after EVOOE treatment, Figures S2 and S3), we tried to discriminate between the protective or not-protective forms of autophagy [57]. This type of cell death can exert opposite effects on cancer cells depending on the cellular context and tumor progression. In particular, the induction of protective autophagy results in enhancing cancer cell survival since it confers resistance to the treatment and increases apoptosis when blocked. Instead, triggering a not-protective form of autophagy can be associated, for example, with the activation of cellular differentiation or senescence, which may contrast with uncontrolled cell growth [58,59].

Thus, we pre-treated cells with chloroquine, a pharmacological inhibitor of autophagic flux. In the case of “protective” autophagy, following the inhibition of the autophagic flux with chloroquine, the treatment with EVOOE should result in increased cytotoxicity; alternatively, in the presence of “not-protective” autophagy, chloroquine inhibition would result in no significant change in the cytotoxic effect of EVOOE. As shown in Figure 4d, after the pre-treatment of RT112 cells with chloroquine, the subsequent addition of EVOOE (CQ + EVOOE) failed to significantly reduce cell viability compared to EVOOE mono-treatment. Therefore, we concluded that EVOOE induced a not-protective autophagic phenotype in RT112 cells.

### 3.4. Pro-Apoptotic Effects of Extra Virgin Olive Oil Phenolic Extract in J82 Cell Line

**Figure 5:**
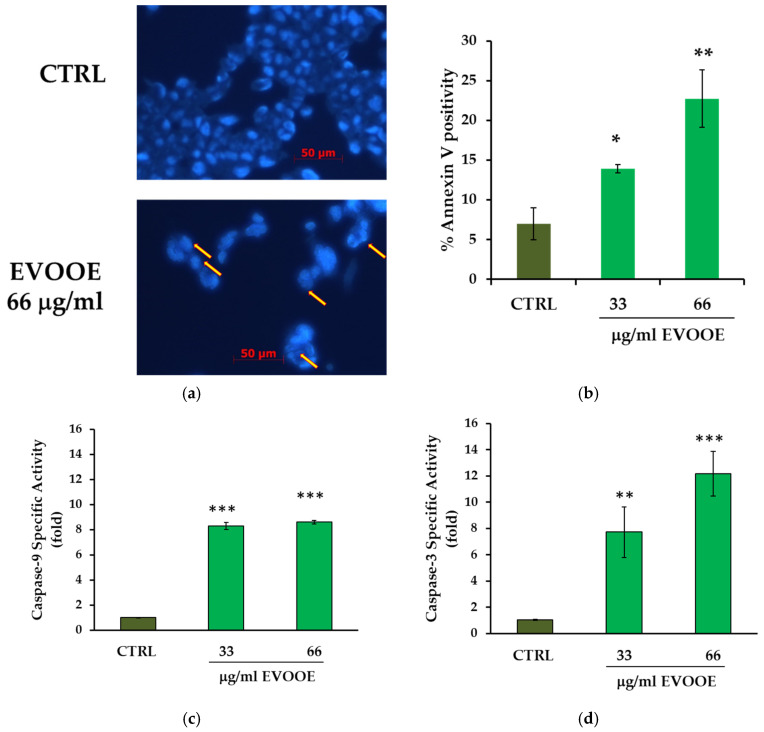
EVOOE stimulation induces apoptosis in J82 cells. (**a**) Representative images of nuclear staining with Hoechst dye of J82 cells untreated (top) and treated with EVOOE (bottom). Fluorescent microscopy was employed to visualize and photograph cells in a DAPI filter with 200× magnification. The arrows indicate the apoptotic bodies identified. (**b**) Annexin-V positivity to assess PS externalization was measured by cytofluorimetric analysis in cells treated for 15 h. (**c**) The proteolytic activity of caspase-9 and (**d**) caspase-3 (nmol AFC/min/mg protein) was measured after 6 h of treatment. Bar graphs represent means ± s.d. derived from three separate experiments. Symbols indicate significance: *p* < 0.05 (*), *p* < 0.005 (**), and *p* < 0.0001 (***) with respect to CTRL.

Assuming that the rapid and extensive reduction of cell viability induced by EVOOE in J82 cells was due to the induction of apoptotic cell death, as suggested by microscopy observation, the presence of apoptotic bodies was initially evaluated. As shown in Figure 5a, EVOOE strongly induced apoptosis, as evidenced by the presence of numerous apoptotic bodies after nuclei staining. To confirm this observation, other apoptotic assays were performed. The PS externalization was assessed through the cytofluorimetric assay using the binding of Annexin V. We observed that EVOOE at 33 and 66 μg/mL concentrations efficiently and significantly induced apoptosis, in a dose-dependent manner (Figure 5b), without evidence of necrosis. Subsequently, we verified the activation of caspases 9 and 3. The former is the initiator caspase in the intrinsic apoptotic pathway that proceeds with the subsequent activation of effector caspases, such as caspase-3, responsible for the cleavage of substrates, like poly (ADP-ribose) polymerase (PARP) [60]. As reported in Figure 5c, a strong increase in caspase-9 activity of approximately 8.3-fold and 8.6-fold was detected at the indicated concentrations. This increase is paralleled with Annexin V data. Similarly, EVOOE induced a significant increase in caspase-3 activity of 7.7- and 12.2-fold in J82 cells treated with 33 μg/mL and 66 μg/mL, respectively (Figure 5d).

### 3.5. Antioxidant Activity of EVOOE in Bladder Cancer Cell Lines

**Figure 6:**
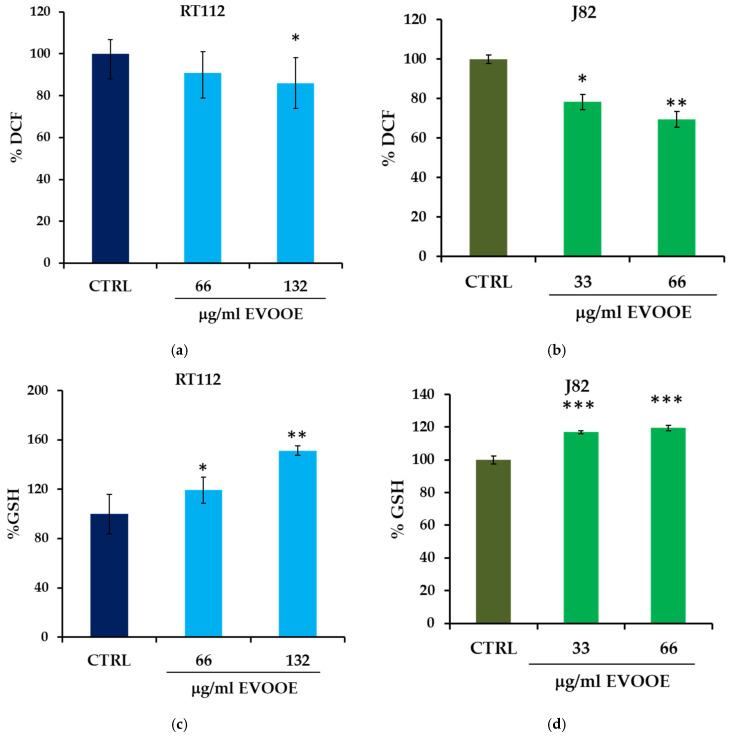
Antioxidant effect of EVOOE in RT112 and J82 cells. Intracellular ROS were measured as DCF fluorescence; RT112 (**a**) and J82 (**b**) cells were stimulated with the indicated concentration of EVOOE for 30 min. Bar graphs represent means of three separate experiments ± s.e. Symbols indicate significance: *p* < 0.05 (*) and *p* < 0.005 (**) with respect to CTRL. GSH content was measured after the treatment of RT112 (**c**) and J82 (**d**) cells with the indicated concentration of the extract for 3 h and compared to untreated samples. Bar graphs represent means of three separate experiments ± s.e. Symbols indicate significance: *p* < 0.05 (*), *p* < 0.005 (**), and *p* < 0.0001 (***) with respect to CTRL.

The antioxidant effect of EVOOE was assessed by measuring its capacity to reduce intracellular ROS. RT112 and J82 cells treated for 30 min with different concentrations of EVOOE resulted in a significant and dose-dependent reduction of intracellular ROS, highest in the J82 cell line, where at 66 μg/mL concentration of EVOOE, ROS decreased by about 30% (Figure 6a,b). We also measured the levels of GSH, a major antioxidant involved in the removal of ROS. In parallel with ROS reduction, a significant increase in GSH content in both cell lines was determined (Figure 6c,d).

**Figure 7:**
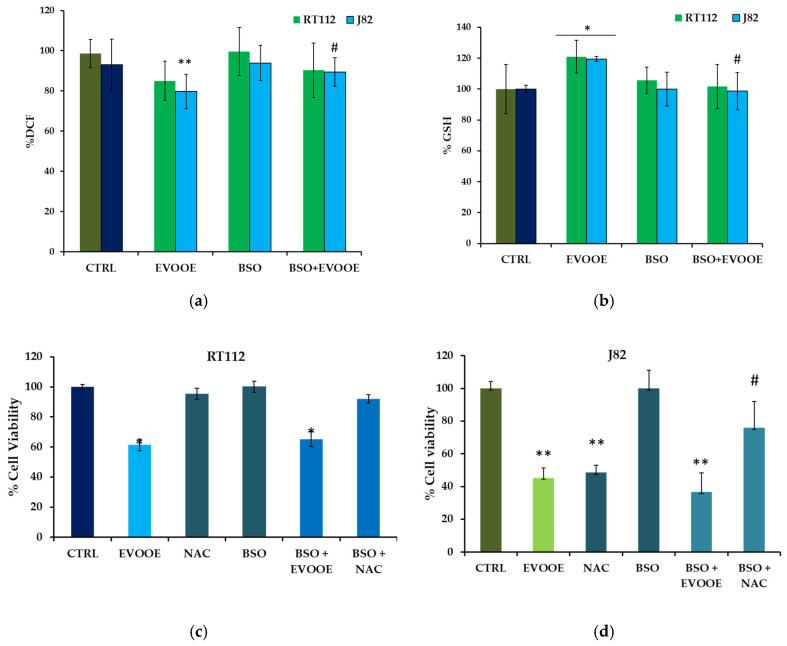
Absence of correlation between antioxidant and anti-proliferative effect induced by EVOOE in bladder cancer cell lines. RT112 and J82 cells were pre-treated for 3 h with BSO (0.5 mM) and subsequently with 66 mg/mL of extract for 3 h to detect GSH content (**a**) and for 30 min to measure intracellular ROS (**b**). RT112 (**c**) and J82 (**d**) cells were pre-treated for 3 h with BSO (0.5 mM) and subsequently with 132 mg/mL and 66 mg/mL of EVOOE, respectively, or NAC (60 mM) for 24 h. Cell viability was assessed by Crystal Violet assay, as reported in the Materials and Methods section. Bar graphs represent the mean of three experiments (±s.e.). Symbols indicate significance: *p* < 0.05 (*), *p* < 0.005 (**) with respect to CTRL In panel (**a**) *p* < 0.05 (#) with respect to EVOOE single treatment and in (**d**) *p* < 0.05 (#) with respect to NAC treatment.

To deepen the mechanism of EVOOE antioxidant response in RT112 and J82 cells, we employed two modulators of GSH synthesis, the GSH precursor *N*-acetylcysteine (NAC) and the GSH inhibitor buthionine sulfoximine (BSO). The latter reduced GSH levels in EVOOE-treated cells to values similar to control (Figure 7a) in both cell lines and counteracted the capacity of EVOOE to reduce intracellular ROS (Figure 7b). NAC (60 μM) was able to increase intracellular GSH levels by about 40% in RT112 and 55% in J82 cells. Comparing the effects on cell viability induced by EVOOE, we observed that in RT112, the treatment with NAC, despite the increment of GSH, did not reduce cell viability (Figure 7c). Moreover, in pre-incubating cells with BSO, the effects on cell viability induced by EVOOE were unchanged (Figure 7c). This result suggested that the action exerted by EVOOE on RT112 cells was independent of its antioxidant properties. Instead, treating J82 cells with NAC, we observed a decrease in cell viability that is comparable to the effect of EVOOE (Figure 7d), indicating a potential role of GSH in this process. In this cell model, the pre-incubation with BSO significantly reduced the effect of NAC (Figure 7d), confirming the possible role of GSH in the reduction of cell viability. However, the pre-incubation with BSO did not affect the EVOOE-induced anti-proliferative effect, suggesting that the activity exerted by the phenolic extract was independent of GSH modulation.

To further investigate the possible correlation between the antioxidant and the anti-proliferative effect induced by EVOOE stimulation in J82 cells, we compared the activity exerted by EVOOE with those induced by some of the most known phenolic antioxidants. Table 1 reports the data obtained stimulating J82 cells with 5 μg/mL (*w*/*v*) of a phenolic extract obtained from green tea (highly rich in polyphenols) and with 30 μM of pure molecules belonging to the polyphenols family, quercetin, gallic acid, myricetin, kaempferol, and rutin. The applied concentrations were extrapolated to be in the range of the GAE calculated for EVOOE (33 μg/mL corresponds to 31.4 μM GAE). All the extracts and the molecules tested strongly diminished intracellular ROS levels, but only rutin, kaempferol (slightly), and quercetin (strongly) reduced cell viability. These data confirm that the antioxidant and anti-proliferative effects induced by EVOOE in this cell model were not functionally correlated.

## 4. Discussion

In the present study, we demonstrated that the phenolic extract obtained from an Italian blend of EVOO possesses a chemopreventive potential and can induce different autophagy and apoptosis in human bladder cancer cell lines depending on tumor progression.

Our data stimulate several questions that crowd and seek an answer. How does the mixture of phenolic compounds in EVOO activate different anti-proliferative pathways? Are polyphenols able to induce autophagy and/or apoptosis in many tumor cells [61,62,63]? These two different forms of programmed cell death control important pathways regulating cell survival and cell death and can be closely interconnected. Autophagy is a conserved biological process that is essential in maintaining homeostasis and metabolic balance. It is an intracellular catabolic process that degrades and recycles misfolded, damaged, or aggregated proteins and whole organelles. Autophagy can have an anti-carcinogenic role in normal cells, but aberrations in its pathways can impact gene derangements, cell metabolism, immune surveillance, metastasis, and tumor drug resistance [58,64,65,66]. Instead, apoptosis is a genetically programmed form of cell death triggered by diverse stimuli, both extracellular signals and intracellular events. Induction of apoptosis results in a cascade of biochemical events resulting in blebbing, cell shrinkage, nuclear fragmentation, DNA fragmentation, and finally, death [67,68].

We believe that the different response to the EVOOE treatment needs to be found in the differences between the two cell lines employed. Höhn et al. [69], studying the mechanisms responsible for the different acquired cisplatin resistance of urothelial carcinoma cells, performed a quantitative real-time PCR array to comparatively analyze the mRNA expression of several genes in RT112 and J82 cells. The results revealed cell type-specific differences in the basal mRNA expression; in particular, among others, a significantly stronger mRNA expression of Calpain, p53, Caspase 6, and ERBB2 was detected in RT112 compared to J82 cells. Instead, in the latter, an enhanced expression of MT1A, XAF1, BCL2, and HMOX1 compared to RT112 cells was revealed. Looking in this direction, we are carrying out a mutational analysis of RT112 and J82 cell lines using an Ion Ampliseq Cancer HotSpot panel, and we found in the RT112 cell line mutated variants of phosphatidylinositol 3-kinase (PI_3_KCA), KDR, APC, MET, p53 genes. When completed, these data may help to decipher the key pathways triggered by EVOOE and responsible for the differential phenotypic response to the treatment (data in progress), supporting the hypothesis that highly expressed apoptosis- or autophagy-associated proteins and signaling pathways can be modulated by phenolic compounds. This assumption finds its rationale in the observation that EVOOE consists of molecules functionally pleiotropic, possessing multiple intracellular targets and, therefore, able to affect different cell signaling processes [70].

An additional open question regards the consequence of a “non-protective autophagy” induced by EVOOE in RT112 cells. This form of autophagy, when inhibited, neither sensitizes nor protects the tumor cell from exogenous stress (e.g., radiation and chemotherapeutic drugs) [57]. However, it is known that when the intensity and duration of autophagy exceed the threshold required for cell survival, autophagic cell death is activated [71]. Starting from this consideration, we are evaluating the effect of EVOOE for longer times, and preliminary data using EVOOE at 132 μg/mL for 72 h showed an increase of the apoptotic markers (caspase-3 and Annexin-V positivity; data in progress). Therefore, we suppose that at lower concentrations and/or shorter times (24 h), EVOOE is unable to pass the threshold necessary to induce cell death, driving the cells into the limbo of autophagy, a condition that can evolve in opposite directions: protecting the cancer cells or killing them, depending on the persistence of the external treatment [58]. Future studies in this direction will help to better define this hypothesis.

Although it is generally assumed that phenols provide health benefits mainly because of their antioxidant activity [26,72,73], the results presented here suggest that, in the case of EVOOE, no clear correlation exists between the antioxidant and the anti-proliferative effects induced in RT112 and J82 cells. As commented above, EVOO polyphenols can modulate several intracellular signals resulting in beneficial effects that are not necessarily interconnected. Concerning the antioxidant capacity characterized in this work, we hypothesized that EVOOE acts mainly through the induction of GSH synthesis. In fact, pre-treating cells with BSO, which is an inhibitor of γ-glutamylcysteine synthetase (γGCS), a key enzyme in GSH biosynthesis, the EVOOE no longer causes an increase in intracellular GSH, and the ROS reduction is weakened (Figure 7). It is highly possible that polyphenols from EVOOE, similar to those from other sources [29,74,75], can modulate transcription factors involved in the expression of critical genes for GSH synthesis. As an example, the transcriptional control of the γGCS catalytic subunit is regulated at the 5’ region where several response elements, including AP-1 sites, one NF-κB site, and several AREs/EpREs are present [74,76]. In this context, future studies will be aimed at investigating the lack of association between antioxidant and antitumor activities such as redox-silent. Redox-silent vitamin E analogs have been indicated as able to induce selective cancer cell death and tumor growth suppression, acting synergistically on cellular organelles (e.g., mitochondria) and triggering their apoptogenic potential [77]. We extensively reviewed the controversial topic of the putative antioxidant effects of phytochemicals in cancer [70], ending up with the conclusion that several confounding factors can be generated by the different doses employed, pharmacological vs. nutritional, and by the diffuse but incorrect concept that cancer treatment and cancer prevention overlap.

Further, we hypothesized that different compounds within EVOOE, such as hydroxytyrosol, tyrosol, oleocanthal, oleuropein, and ligstroside (the latter two in form of aglycone), assessed by HPLC analysis, may contribute to the biological effects reported here. In fact, some of the compounds identified, such as hydroxytyrosol and tyrosol, are widely reported in the literature for their potential therapeutic effects both in vivo and in vitro. Besides their antioxidant properties, hydroxytyrosol and tyrosol are known to exert anticancer activity, improve endothelial dysfunction and lipid profiles, as well as reduce inflammation, oxidative stress, and neurodegeneration [15,78,79,80]. The protective effect is mediated, in addition to the antioxidant and scavenging properties, through the regulation of the intracellular signaling pathway that results in the cellular response to stress and pro-inflammatory factors [15] and ligstroside, whose anti-proliferative effects have been shown in human liver, colon, and breast cancer cell lines [81,82]. Moreover, there has been increasing evidence that oleuropein, another compound present in EVOOE, may play a role in chemoprevention, which has been assessed in animal models [83,84]. The health-promoting properties of these compounds encourage further research to understand their role in EVOOE.

In light of these assumptions, a more targeted study may be required to identify the key compounds that are responsible for the biological activity of EVOOE and determine whether they act synergistically and/or additively.

A key question is how and if it is possible to translate in vivo the effectiveness of EVOOE. Once ingested within the diet, EVOO polyphenols are exposed to extensive metabolism in the human body. They are found in the urine and plasma mainly as conjugated forms, such as glucuronides, sulfates, and methylates and the bioavailability of the aglycones is poor with respect to their metabolites [15,85]. The pairing between the absorption and metabolism of polyphenols versus their anticancer efficacy can be assessed in adequate animal models to envisage the translation from basic research to the clinic. The use of in vivo models will allow for a comprehensive study of the chemopreventive role of EVOOE in multistep cascades of carcinogenesis progression in the bladder, also enabling the investigation of premalignant phases of the disease that are not clinically encountered [86]. The *N*-Butyl-*N*-(4-hydroxybutyl)nitrosamine (BBN)-induced rodent tumors [87] recapitulate the human disease and can be employed to study the early phases of bladder carcinogenesis. Alternatively, bladder cancer GEM (genetically engineered mouse) models that use the mouse Uroplakin II (UpkII) promoter (proteins constituting the major differentiation products of the urothelium) [88] can be employed to assess the efficacy of preventive or therapeutic strategies targeting different stages of bladder cancer development. An obvious corollary of this reasoning is that EVOOE cannot certainly be intended as a “functional food” and be administered at nutritional doses. It is rational to predict its use in pre-clinical studies at pharmacological or sub-pharmacological ones and, possibly, in association with other conventional chemotherapeutic drugs. As we discussed elsewhere, the grey zone between prevention and therapy and nutritional vs. pharmacological doses must always be kept in mind in considering the pros and cons of the beneficial effects of polyphenols against chronic and degenerative pathologies [70].

Finally, it is mandatory to design new and appropriate controlled release systems to increase their bioavailability. These investigations will allow the accumulation of data and information of fundamental importance to plan future human trials.

## 5. Conclusions

In the present work, we studied the role of EVOOE in different stages of bladder cancer progression. The results obtained suggested that EVOOE induces different responses depending on the tumor staging. In the RT112 cell line, representative of low-grade bladder cancer, the phenolic extract induces an autophagic process, pausing cell growth. In J82 cells, representative of a high-grade stage, EVOOE stimulates massive apoptosis. Moreover, the EVOOE exerts in both cancer cell lines antioxidant effects, reducing ROS levels and increasing intracellular GSH levels. However, there was no clear correlation between the antioxidant and the anti-proliferative capacities of EVOOE. We hypothesized that phenolic compounds in EVOOE possess pleiotropic activities that intercept different pathways resulting in anti-proliferative effects. Future investigations will deepen the fine mechanisms underlying the EVOOE anti-proliferative effect and the different responses depending on bladder tumor staging.

## Figures and Tables

**Table 1:** Reduction of intracellular ROS and cell death induced by EVOOE in J82 cells.

Samples	% DCF Reduction (30 min)	% Cell Death (24 h)
Quercetin 30 μM	34.3 ± 3.2 ^#^	31.1 ± 1.2 **
Gallic Acid 30 μM	49.9 ± 4.0 ^#^	1.8 ± 2.0
Myricetin 30 μM	46.0 ± 9.6 ^#^	1.7 ± 5.7
Kaempferol 30 μM	40.6 ± 8.1 ^#^	12.9 ± 4.5 *
Rutin 30 μM	48.9 ± 5.3 ^#^	15.1 ± 4.5 *
Green Tea 5 μg/mL	51.0 ± 6.2 ^#^	0.1 ± 2.4
EVOOE 33 μg/mL	57.9 ± 4.0 ^#^	43.7 ± 2.8 ***

^#^*p* < 0.05, * *p* < 0.05, ** *p* < 0.005 and *** *p* < 0.0001 respect to untreated cells.

## Data Availability

The data presented in this study are available on request from the corresponding author.

## Supplementary Materials

The following supporting information can be downloaded at: https://www.mdpi.com/article/10.3390/nu15010182/s1, Table S1: Hydrogen peroxide measured in culture medium by FOX assay method; Figure S1: EVOOE slightly reduces cell viability in RT112 cell line at 24, 48 and 72 h; Figure S2: EVOOE did not induce apoptosis in RT112 cells; Figure S3: EVOOE did not affect RT112 cell cycle.
