# Supplementary material for: Phenolic Extract from Extra Virgin Olive Oil Induces Different Anti-Proliferative Pathways in Human Bladder Cancer Cell Lines
Source: Nutrients. 2022 Dec 30;15(1):182. doi: 10.3390/nu15010182 (PMC9823665; doi:10.3390/nu15010182)
Supplement: Supplementary file 1 [file nutrients-15-00182-s001.zip › nutrients-2104205-supplementary.pdf]

Supplementary data

Phenolic Extract from Extra Virgin Olive Oil Induces Different Anti-Proliferative Pathways in Human Bladder Cancer Cell Lines

Carmela Spagnuolo *, Stefania Moccia, Idolo Tedesco, Giuseppina Crescente, Maria Grazia Volpe, Maria Russo and Gian Luigi Russo

Table S1

Hydrogen peroxide measured in culture medium by FOX assay method

SAMPLES	560nm	H_2O_2 microM
MEM+EVOOE66	0.001	-104
MEM+EVOOE132	0.003	-100
RPMI+EVOOE66	0.017	-70
RPMI+EVOOE132	0.025	-52
H2O2 1microM	0.16	1
H2O2 10microM	0.273	10
H2O2 100microM	0.647	100

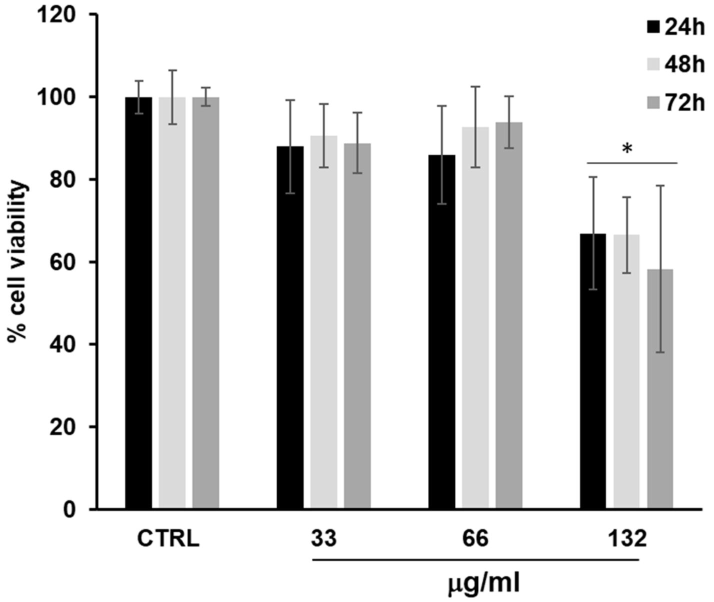

Figure S1. EVOOE slightly reduces cell viability in RT112 cell line. RT112 cells were treated for 24, 48 and 72 h with the indicated concentrations (w/V) of EVOOE. Cell viability was evaluated by Crystal Violet assay. Bar graphs represent the mean of three experiments (± s.e.). Symbols indicate significance: $p<0.05$ (*) respect to CTRL (DMSO-treated cells).

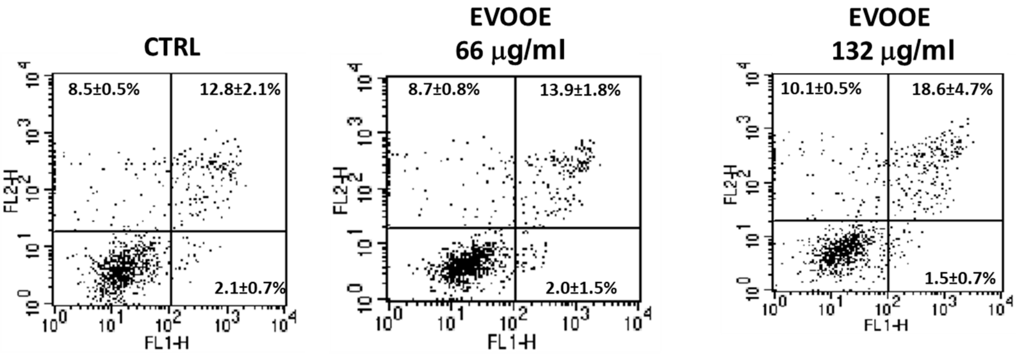

Figure S2. EVOOE did not induce apoptosis in RT112 cells. Representative images of dot-plot obtained by cytofluorimetric analysis are reported. Cells (1.5 × 10⁶/mL) were treated with indicated concentrations of EVOOE for 24 h and then annexin V and propidium iodide positivity were evaluated by flow cytometry (Facscalibur; BD Biosciences, Sparks, MD, USA). Data were analysed by BD CellQuest software and numbers in the quadrant indicate the percentage of cells ± S.D.

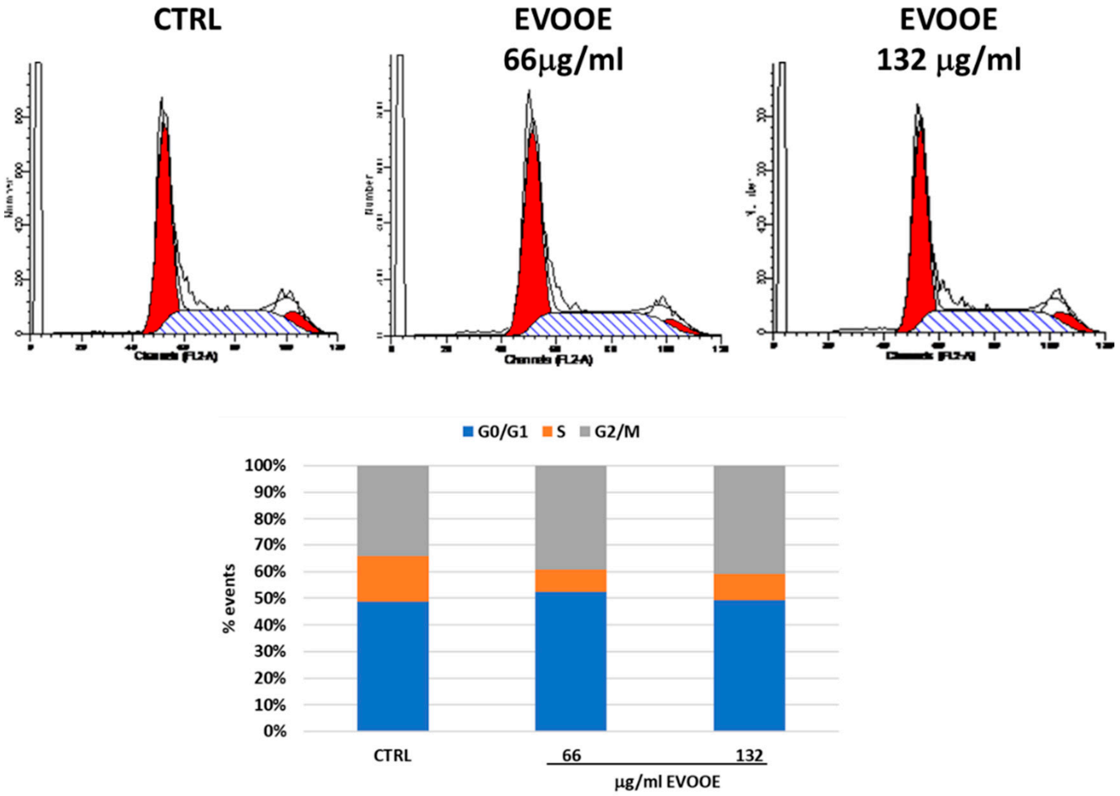

Figure S3. EVOOE did not affect RT112 cell cycle. RT112 cells (1.5 × 10⁶/mL) were treated for 48 h with EVOOE, harvested, fixed with ice-cold 70% ethanol, washed in PBS, and stained using 50 µg/mL propidium iodide in the presence of 100 µg/mL of DNAse-free RNAase A for 1 h at 37 °C in the dark. After the staining procedure, cells were analyzed by flow cytometry (Facscalibur; BD Biosciences) and the DNA content was quantified using ModFit LT software 3.0 (Verity Software

House, Inc., Topsham, ME, USA) to calculate the percentage of diploid cell distribution in the different phases of the cell cycle.
